# Process evaluation of a community-based intervention promoting multiple maternal and neonatal care practices in rural Nepal

**DOI:** 10.1186/1471-2393-10-31

**Published:** 2010-06-07

**Authors:** Robert A McPherson, Jyotsna Tamang, Stephen Hodgins, Laxmi R Pathak, Ram C Silwal, Abdullah H Baqui, Peter J Winch

**Affiliations:** 1 Department of International Health, Johns Hopkins Bloomberg School of Public Health, Baltimore, USA; 2 Centre for Research on Environment Health and Population Activities (CREHPA), Kathmandu, Nepal; 3 Nepal Family Health Program, Kathmandu, Nepal; 4 Ministry of Health and Population, Government of Nepal, Kathmandu, Nepal

## Abstract

**Background:**

The challenge of delivering multiple, complex messages to promote maternal and newborn health in the *terai *region of Nepal was addressed through training Female Community Health Volunteers (FCHVs) to counsel pregnant women and their families using a flipchart and a pictorial booklet that was distributed to clients. The booklet consists of illustrated messages presented on postcard-sized laminated cards that are joined by a ring. Pregnant women were encouraged to discuss booklet content with their families.

**Methods:**

We examined use of the booklet and factors affecting adoption of practices through semi-structured interviews with district and community-level government health personnel, staff from the Nepal Family Health Program, FCHVs, recently delivered women and their husbands and mothers-in-law.

**Results:**

The booklet is shared among household members, promotes discussion, and is referred to when questions arise or during emergencies. Booklet cards on danger signs and nutritious foods are particularly well-received. Cards on family planning and certain aspects of birth preparedness generate less interest. Husbands and mothers-in-law control decision-making for maternal and newborn care-seeking and related household-level behaviors.

**Conclusions:**

Interpersonal peer communication through trusted community-level volunteers is an acceptable primary strategy in Nepal for promotion of household-level behaviors. The content and number of messages should be simplified or streamlined before being scaled-up to minimize intervention complexity and redundant communication.

## Background

### Behavior change in maternal and newborn care interventions

Nepal is projected to meet the Millennium Development Goal to reduce under-five mortality by two-thirds by 2015 [1,2]. Child mortality (children aged 12 to 59 months) has declined notably in Nepal over the past 15 years while neonatal mortality has declined more modestly; an apparent reduction in maternal mortality over the same period is practically significant but has borderline statistical significance [1,3].

Improved maternal and newborn health (MNH) outcomes can be achieved by improving quality of and access to services and through positive changes in household practices and care-seeking. In Nepal, the potential impact of strengthened antenatal, delivery and postpartum care in community-level and referral facilities is constrained by a preference for home delivery and poor geographic access to health facilities.

Positive changes in MNH-related household behaviors and care-seeking can be achieved through a variety of strategies. Community mobilization approaches that target influential women in the community have been effective in Nepal [4,5]. Alternatively, antenatal home visits or community meetings offer opportunities to influence MNH practices [6-8]. Messages can be promoted by trained health workers or by community health volunteers. These messages frequently include content such as identification of a birth attendant and health facility, setting aside funds for delivery, and identification of transportation options in case of a maternal emergency [7,8]. Messages increasingly promote essential newborn care and recognition of and appropriate response to danger signs [9,10]. Targets for these messages may include decision-makers such as husbands [11-13] and mothers-in-law [14,15].

Promoting change in MNH behaviors is challenging. Practices advocated by public health programs frequently contradict the cultural logic underlying local practices [16-18]. Another challenge is that the person (or people) responsible for the behavior may vary from one household to another. The locus of decision-making depends on the issue; pregnant or postpartum women may have little decision-making authority. Behavioral recommendations should be tailored for the household context in which they are delivered.

### Promotion of maternal and newborn care preparedness

Beginning in March 2002 the Ministry of Health and Population (MoHP) of the government of Nepal introduced a community-based intervention called *Jeevan Suraksha *(literally "Safe Life", but translated as "Birth Preparedness"). Jeevan Suraksha "provides information about recommended actions to be taken at each stage of normal pregnancy and birth, identifies the danger signs that indicate possible complications, and encourages financial planning for normal births and for possible emergencies" [19].

In late 2005, the MoHP, with support from the USAID-funded Nepal Family Health Program (NFHP), began implementing a modified version of Jeevan Suraksha (hereafter referred to as "the intervention") in 2 districts--Banke and Jhapa. Under this initiative, content from Jeevan Suraksha was integrated into a set of activities including 1) health education and counseling with pregnant women and household decision-makers during the antenatal period, primarily by Female Community Health Volunteers (FCHVs); (2) strengthening existing health services; and 3) postpartum home visits by FCHVs.

The MoHP established the FCHV program in 1988 to strengthen the outreach and coverage of government health services. FCHVs, who are recruited from local communities and are unpaid, are initially oriented through 18 days of basic training. FCHVs are posted in every rural ward in Nepal and are supervised by paid government community health workers; nationally, 51 percent of FCHVs reported that they discuss their work with a supervisor from outside their immediate work area one or more times per year. FCHVs, who typically serve 100-150 households, spend an average of five hours per week conducting health promotion activities, dispensing commodities and practicing limited case-management for simple medical conditions in children such as diarrhea and pneumonia. Twenty-seven percent of rural women in the terai report discussing their pregnancy with a FCHV while pregnant [20].

A key aspect of the intervention was the distribution of a pictorial booklet that promotes key MNH practices to all pregnant women who are registered with FCHVs. Practices promoted in the booklet cover topics that include the following:

1. Frequency and timing of antenatal care and check-up content.

2. Tetanus toxoid vaccination, albendazole and iron during pregnancy.

3. Recognition of and appropriate response to maternal or newborn danger signs.

4. Need for improved diet, rest and reduced workload during pregnancy.

5. Preparation for delivery including arranging for skilled birth attendant, choice of facility, savings for care in case of emergency and arranging emergency transportation.

6. Essential newborn care including drying and wrapping immediately following delivery, immediate breastfeeding, delayed bathing, and hygienic cord care.

7. Postpartum care of mother including examination, rest and nutritious food for mother.

8. Use of modern family planning method to space or limit births.

The booklet, which costs US$ 0.60 to produce, consists of 18 laminated cards joined by a ring. The booklet used in the intervention was a revised version of a booklet that was originally developed by the USAID-funded Maternal and Neonatal Health Program [19].

**Figure 1:**
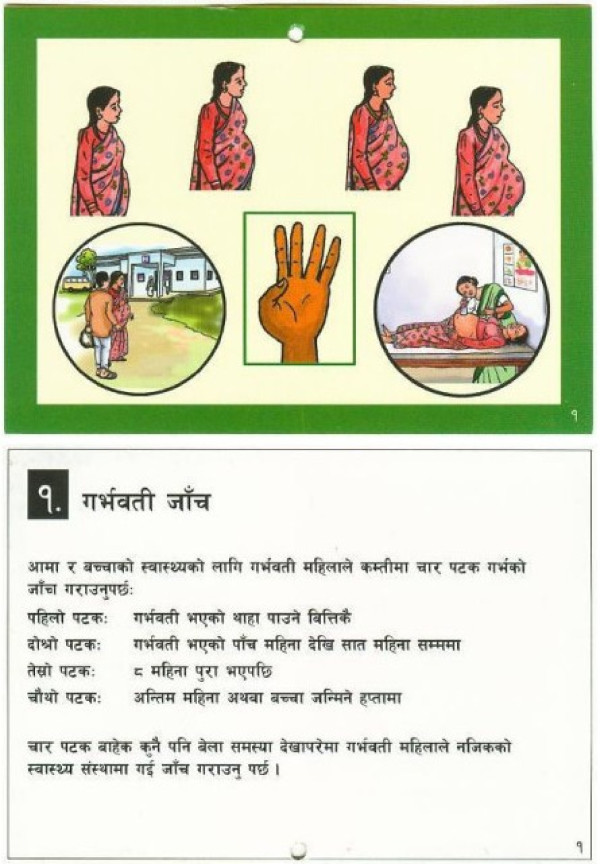
**Booklet card promoting four antenatal visits and accompanying text**.

The booklet, which includes limited text, also relies on images to convey messages. The first card in the booklet is presented in Figure 1, with the illustration on one side of the card and text on the other. This card promotes the need for four antenatal visits, describes recommended timing for each visit, and encourages a pregnant woman with a problem to visit a health facility. Reproductions of all 18 cards can be found in Additional file 1.

**Figure 2:**
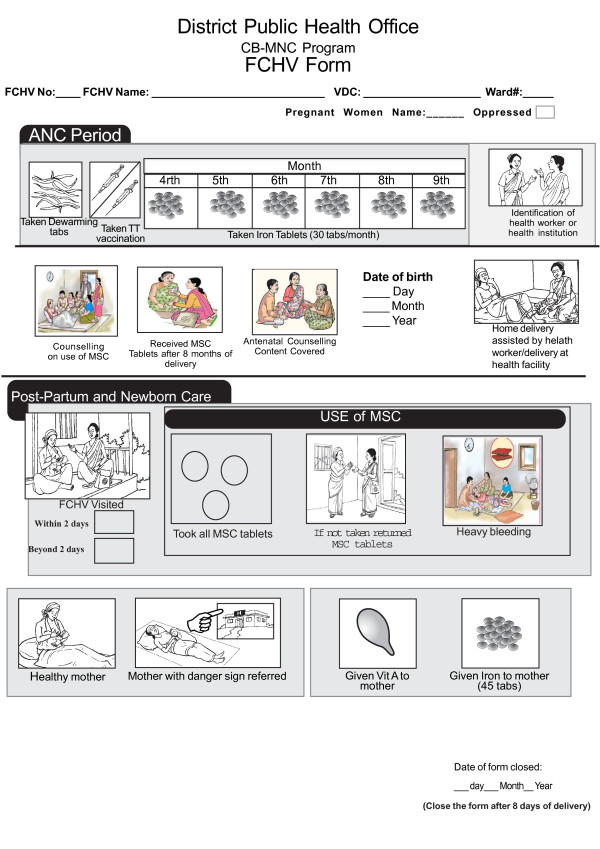
**FCHV pregnancy register**.

Rural areas of Banke and Jhapa districts are served by 665 and 525 FCHVs, respectively. All FCHVs participated in a four-day training in preparation for the intervention. FCHVs were trained to meet four times with each pregnant woman and to visit each woman within three days following her delivery; FCHVs in Jhapa were asked to make an additional visit between four and seven days post-delivery. FCHVs generally contact pregnant women at either the FCHV's or client's home and use a flipchart to counsel all senior household members who are present. During postnatal home visits, FCHVs assess the mother and newborn for danger signs, reinforce counseling messages, and dispense iron-folate and vitamin A to the mother. FCHVs maintain a one-page pregnancy register for each client (an English-language version of the register can be found in Figure 2; FCHVs use a Nepali-language version). The register, flip-chart and booklet contain coordinated content and pictures as a strategy to reinforce messages and FCHV performance. FCHVs attend monthly meetings at their local health facilities and are supervised by staff from health facilities, the District Health Office, and NFHP.

The behavior change strategy of the intervention presumes that FCHVs are effective at promoting care-seeking and desired household practices and that the booklet will facilitate the communication of messages, reinforce the FCHVs' counseling and stimulate intra-household discussion. In this paper we examine these assumptions through a qualitative process evaluation that assesses potential pathways through which the intervention causes change in household practices while exploring factors that affect acceptance of messages and household decision-making. A quantitative description of the effect of the intervention on levels of FCHV activity and household practices is presented elsewhere [21].

## Methods

The process evaluation was conducted in both Banke and Jhapa. Jhapa lies in the eastern terai of Nepal with a total population of 688,109 [22]. The human development index in Jhapa ranks eighteenth among seventy-five districts in Nepal and the adult literacy rate is 62% [23]. Seventy-four percent of pregnant women receive antenatal care from a trained provider while 36% deliver in health facilities [24]. Banke is in the midwestern terai and has a population of 385,840. The human development index in Banke ranks twenty-ninth among districts, the literacy rate is 53%, 77% of pregnant women receive antenatal care and 11% deliver in health facilities.

### Respondents

The evaluation was conducted in three phases. In the first phase, semi-structured interviews were conducted with twenty-one respondents: four central-level NFHP staff, nine district-level government and NFHP health officers (five in Banke and four in Jhapa), and eight staff members (four from each district) who serve in community-level government health facilities and directly supervise FCHVs. In the second phase, semi-structured interviews were conducted with 29 FCHVs. Direct observations of seven antenatal and four postnatal counseling sessions performed by FCHVs were also conducted during this phase. During the third phase, interviews were conducted with 23 women who had delivered within three months of the interview. Other family members who were interviewed during this phase include 14 mothers-in-law and 12 husbands of the mothers who were interviewed. Characteristics of the respondents from Phases 2 and 3 are described in Table 1.

**Table 1:** Characteristics of respondents from Phases 2 and 3.

District	Variable	Phase 2	Phase 3		
	**Respondents**	**FCHVs**	**Mothers**	**Husbands**	**MILs**

	n	17	12	6	6
	
	Age in years (mean (range))	38 (24, 58)	23 (18, 28)	28 (25, 31)	48 (32, 64)
	
Banke	Education				
	None (#)	2	4	1	5
	1-5 years (#)	3	3	0	0
	6-10 years (#)	11	4	4	1
	11+ years (#)	1	1	1	0
	
	Years as FCHV^δ ^(mean (range))	14 (4, 21)			
	
	Primiparous (#)		4		

	n	12	11	6	8
	
	Age in years (mean (range))	34 (22, 56)	22 (17, 30)	29 (23, 35)	54 (45, 67)
	
Jhapa	Education				
	None (#)	1	2	1	7
	1-5 years (#)	2	2	0	1
	6-10 years (#)	9	7	4	0
	11+ years (#)	0	0	1	0
	
	Years as FCHV (mean (range))	7 (1, 17)			
	
	Primiparous (#)		6		

Note: δ = information available from 12 of 17 respondents for "years as FCHV" in Banke.

### Interview content

Research assistants used interview guides that were specific to the three phases to conduct semi-structured interviews and exercises with the booklet cards. A summary of the content of interviews is presented in Table 2.

**Table 2:** Interview content, by phase of evaluation

Phase I	Phase II	Phase III
Perspectives on intervention including perception of booklet and appropriateness of intervention strategy. Impressions regarding FCHV meetings and monitoring and supervision of the intervention. Pile-sorting exercise: Perceptions regarding importance of individual cards in booklet.	Opinion regarding intervention and its effect on maternal/newborn health. FCHV's intervention-related responsibilities and difficulties in fulfilling them. FCHV's experience during visits with clients, use of pregnancy register, and use of the booklet, including her perceptions of its effectiveness. Opinion regarding FCHV training, job aids, FCHV meetings and supervision FCHV receives. FCHV's relationship with community including experiences and difficulties. Preferred format for booklet. Exercises with booklet cards: (1) identification of cards that repeat messages on other cards; (2) pile-sorting exercise to group individual cards in terms of importance as defined by "which cards do you show clients most often (and why)?".	Experiences and problems during latest pregnancy and delivery including newborn care. Comparison of practices during two most recent deliveries and pregnancies (multiparous mothers only). Perception regarding FCHV services including quality of information FCHV provides. How the booklet is used. Intra-household decision-making dynamics regarding maternal and newborn health issues. Preferred format for booklet. Exercises with booklet cards: (1) knowledge of content of cards; (2) choosing the four most useful cards that mother would give to a friend; (3) pile-sorting exercise to identify cards/messages that mother followed.

### Techniques used for exercises with booklet cards

Researchers explored different aspects of respondents' perceptions of the booklet during the interviews. During Phase 1 of the study, the seventeen respondents from district and community-level positions sorted booklet cards into three piles according to their perceived importance. Booklet cards rated as "very important", "somewhat important" and "least important" earned three, two and one point, respectively. A total score was assigned to each card based on the sum across all respondents. FCHV respondents during Phase 2 were asked to sort cards into piles of those they showed clients "most often" and "less often" and then to explain their answers; our team interpreted these data as a behavioral expression of FCHVs' perceptions of the relative importance of the cards. The knowledge of all respondents from Phase 3 regarding card content was assessed by showing respondents each card in turn, asking if they had seen it previously and, if so, what its message was. Respondents' understanding was rated "0" if the message was incorrectly explained or if they hadn't seen the card, "1" if it was partially correct and "2" if it was correctly explained. In order to understand mothers' perceptions of the relative usefulness of the cards, 21 of the 23 mothers serving as respondents during Phase 3 were asked to choose the four most useful cards that they would give to a friend. In another line of questioning intended to explore the concept of "usefulness", mothers were shown the cards sequentially and asked to sort them into two piles: those with messages that they practiced and those that they didn't follow. Respondents who answered negatively were asked to explain why.

We also explored FCHVs' and clients' perceptions regarding the format and overall utility of the pictorial booklet by presenting them with the following three options and asking which would be most effective and why: i) giving flipcharts to FCHVs and nothing to pregnant women; ii) giving flipcharts to the FCHVs and giving pregnant women an inexpensive version of the booklet reproduced in black and white on photocopy paper; and, iii) giving flipcharts to FCHVs and pictorial booklets to pregnant women (as was done in the intervention).

### Selection of respondents

Purposive sampling, guided by explicit selection criteria, was used to select respondents in all three phases of data collection. Four core criteria that guided our sampling in all three phases were to select community-level respondents from (i) diverse geographical locations; (ii) areas with dissimilar representation of ethnic groups; (iii) areas that had not received active monitoring visits from senior NFHP staff or study investigators; and, (iv) areas where FCHVs appeared to be working effectively (as measured by **percentage of expected pregnancies registered by FCHVs**). Our rationale for the latter criterion was that we wanted to observe intervention processes in areas where the intervention was functioning as envisioned. A fifth criterion used to select FCHVs and mothers in Phases 2 and 3 was differential levels of literacy among respondents.

The sample in Phase 1 was composed of (i) all relevant intervention managers at the central and district-level and (ii) community-level health workers who met core criteria described above and who were also well-acquainted with details of the intervention process. Our selection of the sample of FCHVs in Phase 2 was guided by core criteria described above. During the sampling process in Phase 3, after selecting study "areas", members of the research team reviewed pregnancy register forms (maintained by FCHVs) for women who had delivered in the previous one to three months. Forms maintained by FCHVs who served in contiguous catchment areas were not reviewed in order to achieve "spacing" among mothers (i.e., respondents) and the FCHVs who served them. A list was compiled of FCHVs who did not work in adjacent catchment areas and who had submitted completed pregnancy register forms for two or more mothers delivered in the previous one to three months. Research assistants visited these FCHVs and gathered further information on the listed mothers. Mothers were then selected in order to achieve diversity in characteristics that included age, parity, economic status, presence of husband and mother-in-law, ethnicity, literacy, place of delivery, and complications faced during pregnancy or delivery. Mothers-in-laws and husbands of the selected mothers were interviewed if they were present at the time of the mothers' interviews.

### Data collection

Interviews with Kathmandu-based NFHP staff were conducted by one of the authors. All other interviews were conducted by two female research assistants who spoke the local language. Senior members of the research team (Ms. Tamang and Dr. McPherson) trained interviewers in Kathmandu or Banke prior to each phase on interview guide content, interview techniques pertinent to the coming phase, sampling and note-taking procedures, and issues related to confidentiality. All study instruments were pre-tested. During the first several interviews in each phase, research assistants conducted interviews in the presence of study investigators. Following several interviews, the study team met and made changes to the instruments that included adding new questions, changing their order, revising existing questions, and changing the wording in Nepali versions of the instruments for selected questions to more fully realize the intent of the questions as drafted in English. Researchers conducted interviews at respondents' work sites during Phase 1 and at the homes of respondents during Phases 2 and 3. Researchers required one to one and one-half hours to complete interviews during Phase 1 and two and one-half to three hours to complete interviews during Phases 2 and 3; interviews during the latter phases were divided into two parts and conducted either on consecutive days or on the same day with a break between the two parts in order to minimize respondent fatigue. All interviews were tape-recorded with respondent consent. Field notes were recorded in Nepali language; some interviews were later translated verbatim into English from the recording so that study team members not fluent in Nepali could supervise data collection activities and better understand the type and quality of information that was collected. At the conclusion of each phase, interview transcripts were reviewed and major themes and concepts were identified for the purpose of developing codes for organizing and analyzing the data. Data from the first phase were entered into ATLAS-Ti software program for analysis. Data from the second and third phases were manually compiled and tabulated into a matrix according to question and theme. Data were then cross-checked with field notes for verification. Data were read repeatedly to identify patterns and draw conclusions. A draft set of findings was then generated for each phase and circulated among members of the research team for comments and questions prior to finalizing. Findings and conclusions from each phase were used to develop questions for subsequent phases.

### Ethical review

The study documented in this manuscript was approved by the ethics committee of the Nepal Health Research Council.

## Results

### Effect of intervention on FCHVs' responsibilities, attitudes and recognition by community

The introduction of the intervention resulted in an increase in FCHVs' workloads. New responsibilities that were assigned to FCHVs included identifying every pregnant woman in their catchment areas; counseling her and her family members on all messages contained in the booklet during four antenatal encounters while advocating for mothers to receive quality care; maintaining a pregnancy register that documented the status of each woman's care; making at least one home visit to each recently delivered mother; and attending intermittent training functions as well as monthly meetings at the health post. FCHVs reported difficulties finding the time to complete their newly assigned tasks given their personal responsibilities; FCHVs who serve scattered populations or who cross rivers on foot during monsoon to reach clients were especially vocal in this regard.

FCHVs in Nepal are generally recognized and honored by the communities they serve for their contributions. Elderly FCHVs who served as respondents noted that they do not want to retire from their positions due to the appreciation they receive from the community. Some FCHVs stated that now they are trusted more than before the intervention by their clients due to the new services they provide. Other FCHVs expressed the pride they feel in providing women and children with information and services. One FCHV noted that the enhanced supervision that she received under the intervention motivated her to work harder.

### Client perspectives on information sources and role of FCHVs in advocating messages

Mothers, mothers-in-law and almost all husbands who were interviewed perceive information given by their FCHVs to be accurate and trustworthy:

"The information our FCHV gave was very useful. If they give such information to both mothers-in-law and daughters-in-law then it will be easy for us to help our daughters-in-law during their pregnancies. The daughters-in-law must know about danger signs and tell us if they face them." (Mother-in-law, 64 years old)

The intervention partially met demand from household members for information about specific health services. Several respondents said they would have preferred to receive more detailed information on where to go for delivery and how to arrange for transportation. Some mothers mentioned that they would prefer if the FCHV would promote messages from the booklet with their family members more aggressively as it is difficult for mothers to influence senior family members regarding care-related decisions. For example, one mother said she would have preferred that the FCHV convince her mother-in-law to arrange for a skilled birth attendant.

### Intra-household use of booklet and its effect on practices

FCHVs distribute the booklet to pregnant women with the suggestion that they share it and discuss its content with household members and acquaintances. This clearly takes place--all husbands and most mothers-in-law had seen the booklet. All of the interviewed husbands except one reported having discussed the booklet with their wives or other family members. The topics they discussed included danger signs and nutritious food. Several FCHVs reported that their pregnant clients discuss the booklet with their husbands. FCHVs say that women in the community talk about the booklet during their free time and during mothers' group meetings. Pregnant women visit FCHVs and ask them for the booklet and talk to them about their pregnancies.

Among the thirteen multiparous mothers who were interviewed during Phase 3, ten of them stated that they had adopted health practices during their most recent pregnancy that they had not followed during previous pregnancies; all ten respondents stated that the primary source of information influencing these new actions was either the booklet or the FCHV. Five mothers said that it had been decided to delay bathing their newborns based on advice from FCHVs while another mother had read the booklet and made her decision. Two respondents reported that reading the booklet prompted a discussion about where to seek antenatal care while one respondent reported that the booklet had convinced her to save money for the delivery. One husband said that he read the booklet and then helped his wife buy and prepare nutritious food. Another husband mentioned that the next time his wife becomes pregnant he will ask her to do everything written on the booklet and will give her more care and plan to deliver in the hospital.

"I called the doctor when my wife began labor. I gave her hot water and prepared her meals. I also massaged the baby, washed her clothes and prepared hot water for her bath. The FCHV told me to help my wife as much as possible. It is written in the booklet that pregnant women should be taken care of and be loved and that is why I did this." (Husband, 23 years old)

Mothers who had not followed the messages on selected cards provided insight into their reasoning. Some mothers did not have a postnatal check-up because they did not know where to go while others were not permitted to leave their homes before their newborns' *nwaran *(name-giving ceremony). Some respondents did not use contraceptive methods for religious reasons, because their husbands work abroad, or because their menstruation had not resumed following delivery. Although arranging for blood donation is a message promoted in the booklet, many respondents did not act upon this advice. The reality in much of Nepal is that there is little a family can do in this regard due to a lack of blood bank services; if a woman needs a transfusion and if such a service is available, she will receive a transfusion regardless of her family's ability to provide blood.

### Intra-household decision-making and management of health problems

FCHVs in both districts stated that they obtain permission from the mother-in-law or husband before approaching a pregnant woman for the first time. The decision to seek antenatal care is usually taken by the husband and the mother-in-law after discussing with other family members; mothers-in-law appear to make decisions on routine antenatal check-ups while husbands become involved if care is sought for a health problem. These two groups divide responsibility for delivery preparation as well--mothers-in-law generally determine where the delivery will take place, who will attend it, and help save money for the delivery, while husbands often arrange for transportation. Mothers-in-law were found to be the primary decision-maker during delivery and for newborn care practices such as delayed bathing of the newborn.

The combination of booklet content and counseling by the FCHV has become an important resource for some families when maternal or newborn health problems occur. Among six respondents who reported experiencing a problem during pregnancy, two of them first consulted the FCHV or the booklet, while a third contacted her FCHV at the second step.

"My wife experienced severe abdominal pain (during pregnancy). The FCHV was not home so I looked at the booklet and saw that lower abdominal pain is a danger sign. I took her to the clinic in Nepalgunj. There was no doctor there so I took her to the hospital. They gave her medicine and we came home the next day." (Husband, 25 years old)

A similar pattern was seen during delivery; among eight women who faced complications during a home delivery, four sought care first from their FCHV. Among six mothers whose newborns had health problems, one immediately called the FCHV (who referred her to a health facility) while another first consulted the booklet (which provided no information on her newborn's problem) and then proceeded to the health facility. One husband looked at the booklet immediately after the delivery and also when his newborn daughter was sick in order to see if there was any information regarding her symptoms.

### Clients' knowledge and perspectives on relative usefulness and importance of cards

The degree of clients' knowledge of card content was assessed using methods described above. While most mothers and husbands could explain most of the cards correctly or partially correctly, mothers-in-law were less able to do so. Among all respondents, cards on antenatal check-up and nutritious food were best understood while those on delivery preparedness, types of family planning methods available, and newborn danger signs and care were less well understood.

When mothers were asked to choose the four most useful cards that they would give to a friend, the three cards chosen by ten or more respondents were those on antenatal care (i.e., frequency and timing of antenatal care; n = 13), danger signs during delivery (n = 11), and danger signs during pregnancy (n = 10). Other cards that were chosen frequently include those on immediate newborn care practices (e.g., immediate wiping and wrapping, delayed bathing; n = 6), preparations during pregnancy (n = 6) and content of post-delivery care for mother and newborn (n = 6). When asked why they selected the cards they did, respondents noted that if a pregnant woman is aware of danger signs she can seek timely treatment, and that when she visits a health facility for an antenatal or postnatal check-up, apart from the examination, she receives important information from health workers. In response to a separate but related question, husbands reported that the cards they looked at most frequently were those on danger signs and birth preparedness (e.g., saving money, arranging emergency transportation).

Respondents who were interviewed during the first phase perceived the most important messages to be those on danger signs and newborn care. Cards on antenatal care, preparation for delivery and nutritious food were also deemed important. Cards on family planning and certain aspects of birth preparedness (e.g., different ways to save money) were rated least important, in part because these messages are presented on multiple cards that overlap. FCHVs stated that they show cards on danger signs most frequently because these cards are more important than the others.

### Perspectives on structure, content and format of booklet

While respondents from Phase 1 generally agreed that the booklet promotes a large number of messages, some were reluctant to recommend reducing their number because they all seem important. Some program managers stated that some messages are "lost" amidst the large number of messages competing for attention. They noted that reducing the number of messages could reduce the number of required contacts between FCHVs and clients, thereby streamlining the intervention.

"We should change the intervention design to make it more feasible. We should reduce the number of FCHV visits and the number of messages. Some messages are unnecessary--for instance, what is the use of talking to a woman in Jumla (a remote district) about arranging blood donation? There are too many cards on delivery preparation and this confuses the FCHVs." (NFHP staff, Jhapa)

Government and NFHP staff and FCHVs all said that some messages appear on more than one booklet card and are redundant. This not only confuses clients but also causes some FCHVs to skip over cards that repeat information, resulting in the under-emphasis of some important messages. Some FCHVs noted that they discuss cards on antenatal care, family planning and preparations for delivery less than other cards as messages on these topics are repeated across multiple cards.

Clients and FCHVs alike felt that providing pregnant women and their families with some form of written material was crucial--both to refresh clients' memories following counseling sessions as well as to stimulate discussion among other household members. Several mothers noted that any material that is distributed should have pictures so that illiterate women can make use of it. Almost all FCHVs and clients felt that the option of providing pictorial booklets to clients (as was done in the intervention) was preferable to giving clients either an inexpensive version of the booklet reproduced in black and white on photocopy paper or else no communication materials at all. They noted that the colored format of the booklet attracts readers while its laminated, compact construction makes it durable and easy for clients to carry and refer to.

## Discussion

We conducted a process evaluation of a community-based intervention that used interpersonal peer communication, supported by a pictorial booklet and flipchart, to promote behaviors intended to improve maternal and newborn health outcomes. The effectiveness of an earlier version of the pictorial booklet in a terai district in Nepal has been documented elsewhere [7]. Several findings emerged from our study.

### FCHVs and their role in health promotion

FCHVs in Nepal constitute a highly effective cadre. There is an ongoing debate in the literature--and in Nepal as well--regarding the appropriateness of using volunteers in community-level health interventions [25-27], and more specifically concerning their role in newborn care [10,28,29]. Under the intervention addressed here, FCHVs increased their workload and expanded their role and relationship with the community. Independent national surveys of FCHVs from 2005 (prior to intervention) and 2006 (during intervention) document that FCHVs in Banke and Jhapa reported working longer hours following the introduction of the intervention. Despite this enhanced workload, when 2006 survey respondents were asked to compare their current responsibilities with what they are willing to do, most FCHVs indicated they would like to take on additional activities and responsibilities; very few stated that they would like to spend less time on their work [20,30]. The intervention took place in a systems context of strengthened supervision of FCHVs, introduction of regular monthly FCHV meetings, and the provision of educational and record-keeping tools to FCHVs to support their work.

Our research found that the tasks that FCHVs most consistently report performing under the intervention include active identification of pregnant women; provision of counseling at a site convenient for the client and FCHV; dissemination of specific information about maternal and newborn services and household practices, using the flipchart to guide counseling; and, advocacy of proper care for mothers and newborns with their family members.

Members of rural Nepali households were receptive to information provided through the booklet and FCHVs. They trust the messenger and read, discuss, and refer to the booklet at times of need. Some household members want more specific information regarding availability and location of maternal and newborn health services. Mothers want FCHVs to advocate more strongly with household members who hold decision-making power for mothers and newborns to receive quality health services, particularly regarding the importance of SBA-attended delivery. The intervention attempted to convince FCHVs of the validity and importance of the messages that they were to promote, so that FCHVs in turn become ardent local advocates. We did not explore whether FCHVs' failure to advocate more strongly for SBA attendance is related to their lack of belief in the message.

### Use of health education materials in households and communities

Our study explored the dynamics of how the booklet and the information it contains is used within communities and households. The booklet is shared and discussed among household and community members through a number of channels and clearly informs and influences household practices and decision-making. The booklet/FCHV combination is consulted early in the decision-making process in many households in the event of health problems.

Messages that are disseminated through the booklet exert their influence through a hierarchy in which husbands and mothers-in-law are the primary decision-makers. These two groups appear to have somewhat distinct content domains over which they control decision-making. This finding makes it clear that the booklet has different user audiences and suggests that these audiences should be targeted explicitly in future iterations of this intervention. For example, effectiveness may be improved either by refining certain parts of the booklet and tailoring them for specific audiences (e.g., husbands or mothers-in-law) or by developing new tools specific to such groups. Our finding that many mothers-in-law do not fully understand the content of some messages is troubling given their significant decision-making role in maternal-neonatal care. Mothers-in-law in Nepal are mostly illiterate; content oriented to them needs to be self-explanatory without resort to text. Poorly understood illustrations relating to decision-making domains controlled by mothers-in-law should be reviewed, revised, and pre-tested with mothers-in-law.

### Lessons regarding the development of educational materials

Our evaluation yielded valuable insights regarding how educational materials that promote multiple behaviors can best be developed and constructed. The booklet and flipchart used in the intervention present some messages in a repeated manner across cards. This had some negative consequences; some messages deemed redundant by FCHVs and clients are underemphasized or ignored. Repetition of messages consumes valuable space within educational materials and limits the number of messages that can be promoted. The inclusion of "too many messages", a term used by some respondents, also increases the likelihood that messages with marginal importance or application will be included. The potential effectiveness of the booklet and flipchart could be enhanced by eliminating redundancies and removing messages on topics that have not achieved a positive response in the intervention. It may also be more effective to distribute booklet cards incrementally, at relevant stages of pregnancy and post-delivery when they are most salient and immediately actionable; for example, family planning messages could be disseminated during postpartum visits, rather than during pregnancy. This could create a focus for each FCHV-client encounter and encourage clients to review the booklet and discuss its content with family members.

While our results suggest that repetition of messages across cards within the pictorial booklet can be counterproductive, it is important to remember that there is another dimension to "repetition". The health communication literature makes it clear that disseminating a message through multiple channels of communication--e.g., face-to-face, radio, posters--is more effective than communicating it through a single channel. The intervention that we evaluated transmits key messages through multiple channels that include counseling/home visits by FCHVs, booklet cards, posters in health facilities, and counseling by health workers in health facilities. This type of repetition or reinforcement is positive and should be retained as possible.

Clients noted the benefits of receiving written and pictorial materials that reinforce the messages promoted by FCHVs and enable the dissemination of messages among household members who do not participate in counseling. While they stated a preference for the pictorial booklet over simpler materials, it should be noted that significant logistical and supply problems were encountered with the booklet format. For example, the booklet was somewhat bulky and heavy and hard to deliver in quantity to some locations. FCHVs report that cards sometimes become detached from the key ring that binds them and are lost.

The process that is used to develop educational materials that promote multiple messages can play a key role in determining the nature, quality and usefulness of the materials. This process is often implemented through a forum or workshop, attended by a variety of stakeholders, who may represent disparate agendas. The materials are often developed on a consensual basis in order to promote inclusivity and ownership. This approach has clear benefits, particularly in building a sense of ownership, but can produce an unfocussed product. Officials from government and its development partner organizations are generally well-represented in the process, as are trained health workers, especially physicians. However, professionals with two specific skill/experience profiles are often underrepresented or unrepresented in the process: (i) people with strong experience-based knowledge regarding the type of materials that community health workers would find helpful to promote behaviors; and, (ii) professionals with expertise in the design of community-level communication materials such as the booklet. While the process used to develop the booklet bears some resemblance to the process described above, it must be acknowledged that the inclusive process that was followed appears to have resulted in tangible benefits. Widespread buy-in to the booklet by both the government and its partners has resulted in its current use in almost all 75 districts of Nepal.

### Study limitations

Care should be taken in applying the findings of our evaluation beyond the two study districts. The evaluation was carried out in areas of Nepal where the terrain is flat, transportation is relatively good, and a number of health projects have been carried out previously. Other problems with the implementation of the intervention may have been noted if the evaluation had been conducted in a more remote part of Nepal, in an area with different ethnic groups, or in an area where there have been fewer previous health projects. In addition, we did not conduct interviews with a random sample of respondents. It is possible that we did not include respondents from groups that face constraints that prevent them from benefiting from key aspects of the intervention. Finally, we chose to interview community-level respondents from areas where FCHVs appeared to be achieving acceptable coverage of pregnant women in order to evaluate the intervention in areas where it was being implemented successfully. As such, we did not assess intervention processes in areas where FCHVs may not have been working as effectively.

## Conclusions

**Figure 3:**
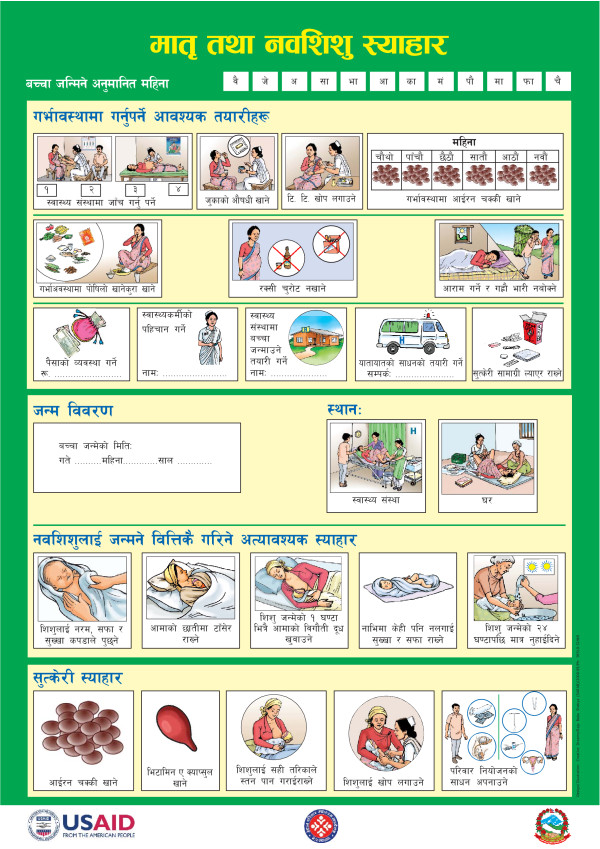
**Revised version of pictorial booklet (page 1)**.

**Figure 4:**
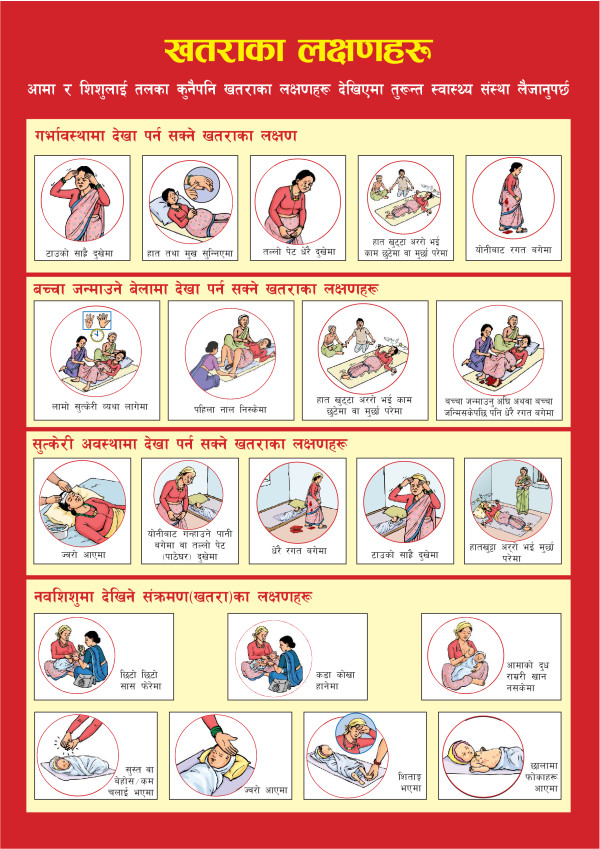
**Revised version of pictorial booklet (page 2)**.

This study has found that interpersonal peer communication through community-level volunteers in Nepal to promote household-level behaviors is acceptable to clients and health workers alike. The content and numbers of messages in the booklet that was studied should be simplified or streamlined to eliminate redundant messages and reduce the complexity of the intervention. While the evidence that we have presented suggests that the booklet was useful and appreciated and that it was part of an effective approach to promoting health practices, we cannot conclusively state that it is better or worse than alternate forms of message dissemination. The booklet represents one possible type of illustrated handout, but we would note that there are other strategies or tools that could be used to disseminate messages that may prove to be equally or more effective at potentially lower cost or with more modest program requirements. Indeed, following the completion of this study, the government and its partners produced a revised one-page (double-sided) version of the pictorial booklet on thick paper in colored format (Figures 3 and 4). This page, which measures approximately 12 by 15 inches, can be produced at one-fifth of the cost of the pictorial booklet and is substantially easier to transport, thus easing some of the logistical problems noted above. Although the new version is easy to hang on the wall and display, most of the text that was present on the pictorial booklet has been eliminated from this new version, which may reduce its effectiveness in reinforcing messages among literate household members who do not attend counseling sessions. It remains to be seen how effectively this revised tool will address the issues that have emerged from our study.

Further studies that would produce useful information include a comparison of alternative combinations of counseling cards for maternal and newborn care to determine the optimum sequence and amount of material. It would also be helpful to determine the best time and approach to present material on postpartum contraception and birth spacing, given that the two cards included in the booklet on family planning were not well received. One approach might be to revise the cards on these topics, plan to add them to the booklet separately during a home visit that would be conducted by the FCHV 3-4 months postpartum, and support this visit with guidelines for associated counseling. Finally, the conduct of a process evaluation of the intervention in areas where it has low coverage remains a research priority.

## Competing interests

The authors declare that they have no competing interests.

## Authors' contributions

RAM, PJW and SH conceptualized the evaluation and designed the protocol for the study. RAM drafted the manuscript with substantive inputs from PJW and SH. JT and RAM designed the data collection instruments, supervised the data collection, coding and analysis, and drafted the initial report on the process evaluation in collaboration with PJW and SH. LRP, RCS and AHB made critical suggestions on the manuscript. All authors read and approved the final manuscript.

## Pre-publication history

The pre-publication history for this paper can be accessed here:

http://www.biomedcentral.com/1471-2393/10/31/prepub

## Supplementary Material

Additional file 1 **Booklet cards**. Reproductions of all 18 booklet cards are included in a zip file. Click here for file

## References

[B1] Thapa S Declining trends of infant, child and under-five mortality in Nepal J Trop Pediatr 2008 54 265 268 10.1093/tropej/fmn011 18310665

[B2] Boerma JT Jennifer B Yohannes K Henrik A Cesar GV Stan B Ties B Betty K Cesar V Mind the gap: equity and trends in coverage of maternal, newborn, and child health services in 54 Countdown countries Lancet 2008 371 1259 1267 10.1016/S0140-6736(08)60560-7 18406860

[B3] MOHP Nepal Demographic and Health Survey 2006 2007 Kathmandu, Nepal: Ministry of Health and Population [Nepal], New ERA, and Macro International Inc

[B4] Morrison J Tamang S Mesko N Osrin D Shrestha B Manandhar M Manandhar D Standing H Costello A Women's health groups to improve perinatal care in rural Nepal BMC Pregnancy Childbirth 2005 5 6 10.1186/1471-2393-5-6 15771772 PMC1079874

[B5] Manandhar DS Osrin D Shrestha BP Mesko N Morrison J Tumbahangphe KM Tamang S Thapa S Shrestha D Thapa B Shrestha JR Wade A Borghi J Standing H Manandhar M Costello AM Effect of a participatory intervention with women's groups on birth outcomes in Nepal: cluster-randomised controlled trial Lancet 2004 364 970 979 10.1016/S0140-6736(04)17021-9 15364188

[B6] Stanton CK Methodological issues in the measurement of birth preparedness in support of safe motherhood Eval Rev 2004 28 179 200 10.1177/0193841X03262577 15130180

[B7] McPherson RA Khadka N Moore JM Sharma M Are birth-preparedness programmes effective? Results from a field trial in Siraha district, Nepal J Health Popul Nutr 2006 24 479 488 17591345 PMC3001152

[B8] Moran AC Sangli G Dineen R Rawlins B Yameogo M Baya B Birth-preparedness for maternal health: findings from Koupela District, Burkina Faso J Health Popul Nutr 2006 24 489 497 17591346 PMC3001153

[B9] Darmstadt GL Kumar V Yadav R Singh V Singh P Mohanty S Baqui AH Bharti N Gupta S Misra RP Awasthi S Singh JV Santosham M Introduction of community-based skin-to-skin care in rural Uttar Pradesh, India J Perinatol 2006 26 597 604 10.1038/sj.jp.7211569 16915302

[B10] Baqui AH El-Arifeen S Darmstadt GL Ahmed S Williams EK Seraji HR Mannan I Rahman SM Shah R Saha SK Syed U Winch PJ Lefevre A Santosham M Black RE Effect of community-based newborn-care intervention package implemented through two service-delivery strategies in Sylhet district, Bangladesh: a cluster-randomised controlled trial Lancet 2008 371 1936 1944 10.1016/S0140-6736(08)60835-1 18539225

[B11] Shefner-Rogers CL Sood S Involving husbands in safe motherhood: effects of the SUAMI SIAGA campaign in Indonesia J Health Commun 2004 9 233 258 10.1080/10810730490447075 15360036

[B12] Carter MW Speizer I Salvadoran fathers' attendance at prenatal care, delivery, and postpartum care Rev Panam Salud Publica 2005 18 149 156 10.1590/S1020-49892005000800001 16269116

[B13] Mullany BC Becker S Hindin MJ The impact of including husbands in antenatal health education services on maternal health practices in urban Nepal: results from a randomized controlled trial Health Educ Res 2007 22 166 176 10.1093/her/cyl060 16855015

[B14] Mesko N Osrin D Tamang S Shrestha BP Manandhar DS Manandhar M Standing H Costello AM Care for perinatal illness in rural Nepal: a descriptive study with cross-sectional and qualitative components BMC Int Health Hum Rights 2003 3 3 10.1186/1472-698X-3-3 12932300 PMC194728

[B15] Masvie H The role of Tamang mothers-in-law in promoting breast feeding in Makwanpur District, Nepal Midwifery 2006 22 23 31 10.1016/j.midw.2005.02.003 15967547

[B16] Winch PJ Alam MA Akther A Afroz D Ali NA Ellis AA Baqui AH Darmstadt GL El Arifeen S Seraji MH Local understandings of vulnerability and protection during the neonatal period in Sylhet District, Bangladesh: a qualitative study Lancet 2005 366 478 485 10.1016/S0140-6736(05)66836-5 16084256

[B17] Nichter M Cultural dimensions of hot, cold and sema in Sinhalese health culture Soc Sci Med 1987 25 377 387 10.1016/0277-9536(87)90276-0 3686087

[B18] Darmstadt GL Saha SK Traditional practice of oil massage of neonates in Bangladesh J Health Popul Nutr 2002 20 184 188 12186200

[B19] Sood S Chandra U Mishra P Neupane S Measuring the Effects of Behavior Change Interventions in Nepal with Population-Based Survey Results 2004 Baltimore MD: Jhpiego

[B20] New_ERA An Analytical Report on National Survey of Female Community Health Volunteers of Nepal: 2006 2007 Kathmandu, Nepal: New ERA, USAID and ORC Macro

[B21] Hodgins S McPherson R Suvedi BK Shrestha RB Silwal RC Ban B Neupane S Baqui AH Testing a scalable community-based approach to improve maternal and neonatal health in rural Nepal J Perinatol 2009 in press 19907428 10.1038/jp.2009.181

[B22] ISRSC District Demographic Profile of Nepal 2002 Kathmandu, Nepal: Informal Sector Research & Study Centre

[B23] UNDP Nepal Human Development Report 2004: Empowerment and Poverty Reduction 2004 Kathmandu, Nepal: UNDP

[B24] VRG Follow-up Survey on CB-MNC Program in Jhapa, Banke and Kanchanpur Districts 2008 Kathmandu, Nepal: Nepal Family Health Program and Valley Research Group

[B25] Frankel S Doggett M-A The Community health worker: effective programmes for developing countries 1992 Oxford; New York: Oxford University Press

[B26] Winch PJ Gilroy KE Wolfheim C Starbuck ES Young MW Walker LD Black RE Intervention models for the management of children with signs of pneumonia or malaria by community health workers Health Policy Plan 2005 20 199 212 10.1093/heapol/czi027 15965032

[B27] Haines A Sanders D Lehmann U Rowe AK Lawn JE Jan S Walker DG Bhutta Z Achieving child survival goals: potential contribution of community health workers Lancet 2007 369 2121 2131 10.1016/S0140-6736(07)60325-0 17586307

[B28] Mannan I Rahman SM Sania A Seraji HR Arifeen SE Winch PJ Darmstadt GL Baqui A Can early postpartum home visits by trained community health workers improve breastfeeding of newborns? J Perinatol 2008 28 632 40 10.1038/jp.2008.64 18596714 PMC2929160

[B29] Baqui AH Arifeen SE Williams EK Ahmed S Mannan I Rahman SM Begum N Seraji HR Winch PJ Santosham M Black RE Darmstadt GL Effectiveness of Home-Based Management of Newborn Infections by Community Health Workers in Rural Bangladesh Pediatr Infect Dis J 2009 28 304 310 10.1097/INF.0b013e31819069e8 19289979 PMC2929171

[B30] New_ERA An Analytical Report on Female Community Health Volunteers (FCHVs) of Nepal: 2005 2006 Kathmandu, Nepal: New ERA, USAID and ORC Macro

