# Supplementary figures and images for: Process evaluation of a community-based intervention promoting multiple maternal and neonatal care practices in rural Nepal
Source: BMC Pregnancy Childbirth. 2010 Jun 7;10:31. doi: 10.1186/1471-2393-10-31 (PMC2898677; doi:10.1186/1471-2393-10-31)

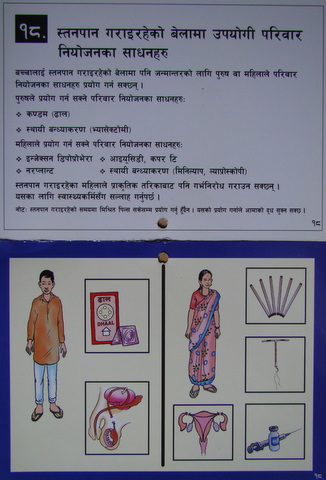

Supplement: Additional file 1 — Booklet cards. Reproductions of all 18 booklet cards are included in a zip file. [file 1471-2393-10-31-S1.ZIP › BPP-card18.JPG]

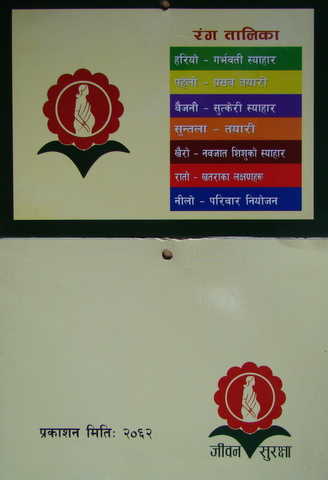

Supplement: Additional file 1 — Booklet cards. Reproductions of all 18 booklet cards are included in a zip file. [file 1471-2393-10-31-S1.ZIP › BPP-cover.JPG]

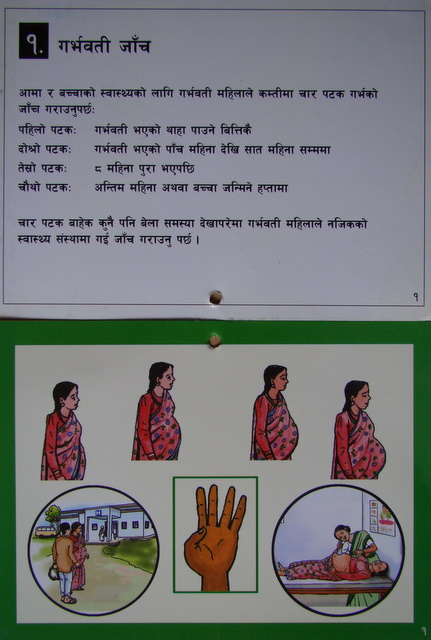

Supplement: Additional file 1 — Booklet cards. Reproductions of all 18 booklet cards are included in a zip file. [file 1471-2393-10-31-S1.ZIP › BPP-card1.JPG]

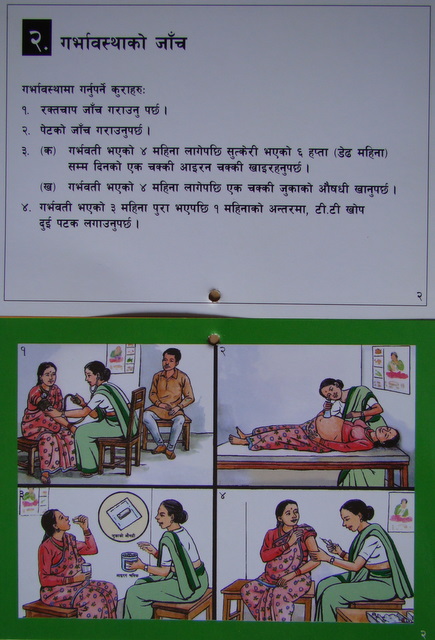

Supplement: Additional file 1 — Booklet cards. Reproductions of all 18 booklet cards are included in a zip file. [file 1471-2393-10-31-S1.ZIP › BPP-card2.JPG]

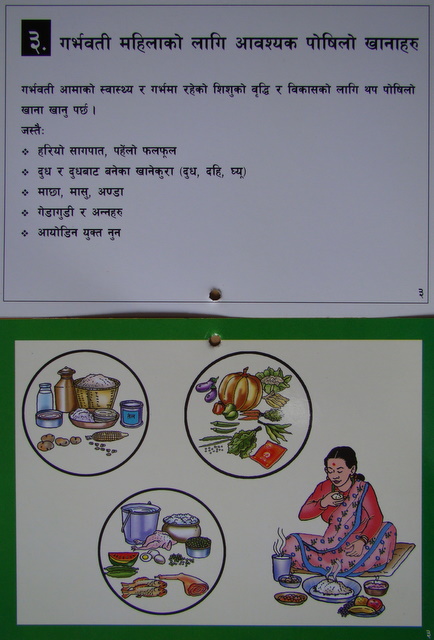

Supplement: Additional file 1 — Booklet cards. Reproductions of all 18 booklet cards are included in a zip file. [file 1471-2393-10-31-S1.ZIP › BPP-card3.JPG]

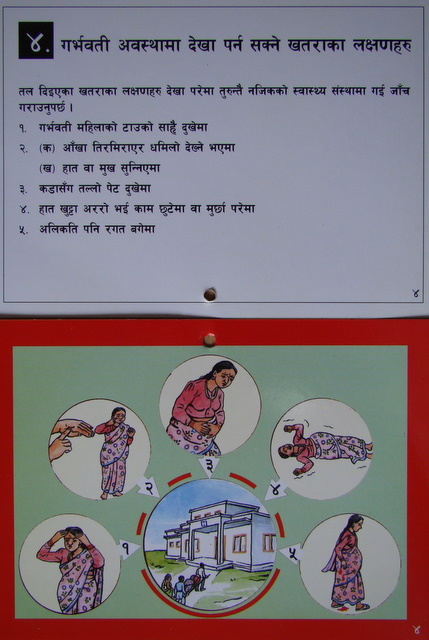

Supplement: Additional file 1 — Booklet cards. Reproductions of all 18 booklet cards are included in a zip file. [file 1471-2393-10-31-S1.ZIP › BPP-card4.JPG]

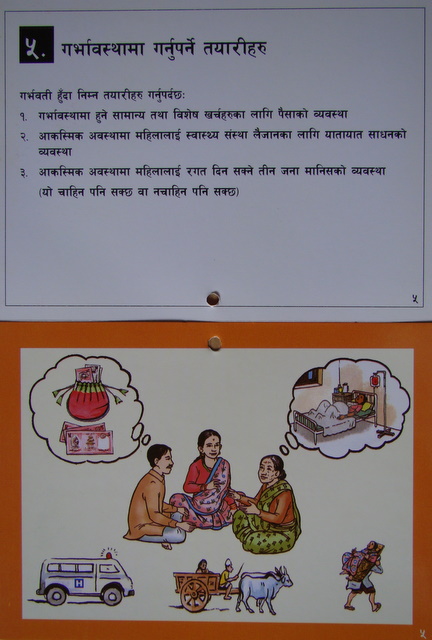

Supplement: Additional file 1 — Booklet cards. Reproductions of all 18 booklet cards are included in a zip file. [file 1471-2393-10-31-S1.ZIP › BPP-card5.JPG]

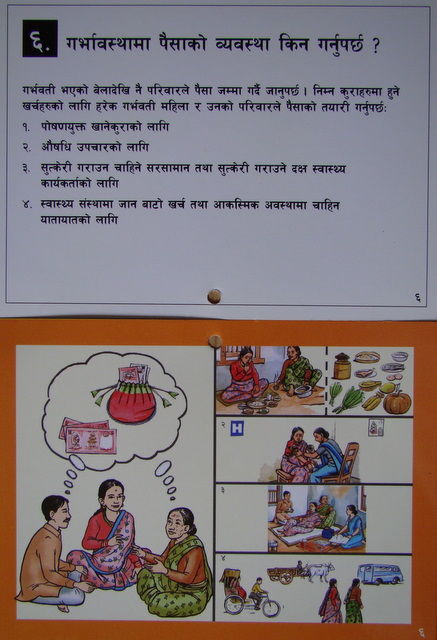

Supplement: Additional file 1 — Booklet cards. Reproductions of all 18 booklet cards are included in a zip file. [file 1471-2393-10-31-S1.ZIP › BPP-card6.JPG]

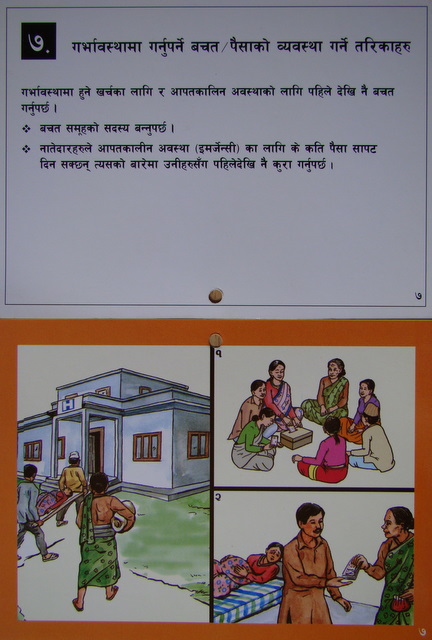

Supplement: Additional file 1 — Booklet cards. Reproductions of all 18 booklet cards are included in a zip file. [file 1471-2393-10-31-S1.ZIP › BPP-card7.JPG]

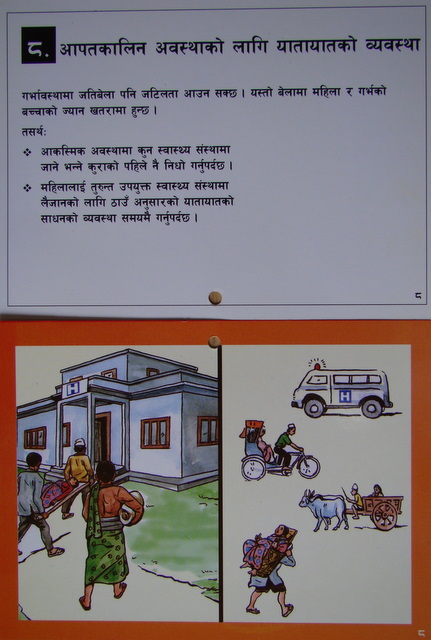

Supplement: Additional file 1 — Booklet cards. Reproductions of all 18 booklet cards are included in a zip file. [file 1471-2393-10-31-S1.ZIP › BPP-card8.JPG]

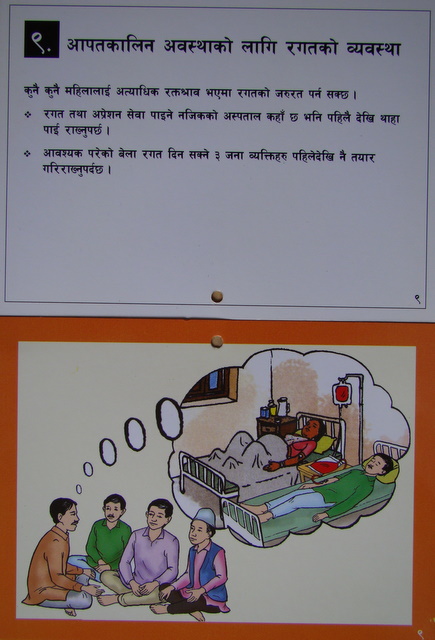

Supplement: Additional file 1 — Booklet cards. Reproductions of all 18 booklet cards are included in a zip file. [file 1471-2393-10-31-S1.ZIP › BPP-card9.JPG]

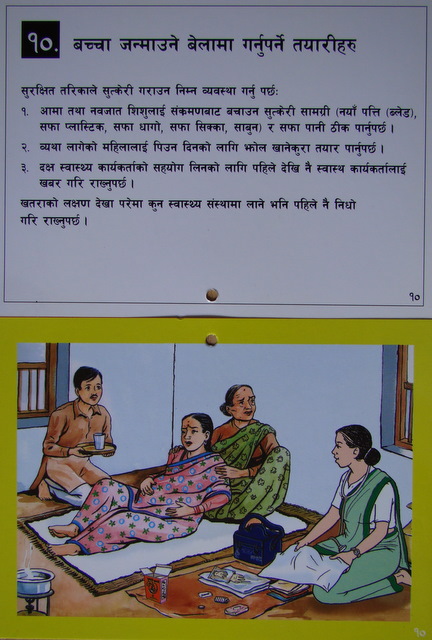

Supplement: Additional file 1 — Booklet cards. Reproductions of all 18 booklet cards are included in a zip file. [file 1471-2393-10-31-S1.ZIP › BPP-card10.JPG]

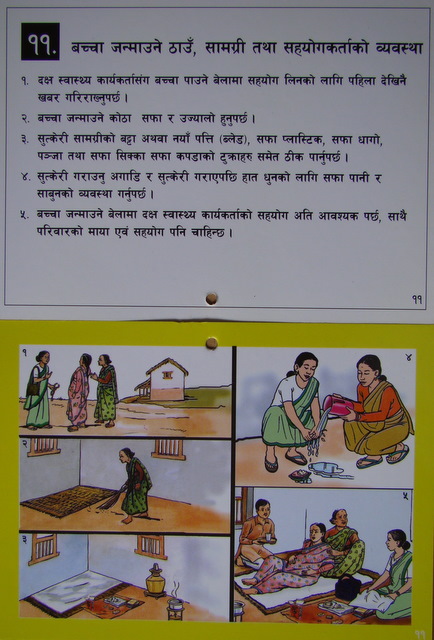

Supplement: Additional file 1 — Booklet cards. Reproductions of all 18 booklet cards are included in a zip file. [file 1471-2393-10-31-S1.ZIP › BPP-card11.JPG]

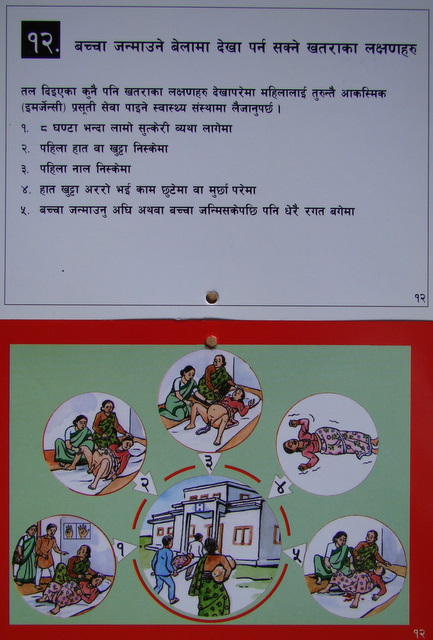

Supplement: Additional file 1 — Booklet cards. Reproductions of all 18 booklet cards are included in a zip file. [file 1471-2393-10-31-S1.ZIP › BPP-card12.JPG]

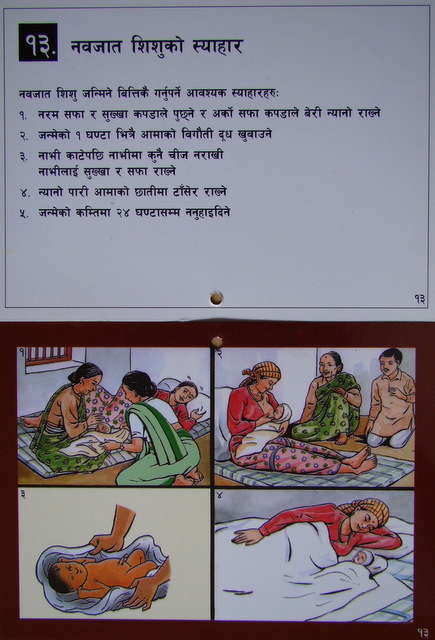

Supplement: Additional file 1 — Booklet cards. Reproductions of all 18 booklet cards are included in a zip file. [file 1471-2393-10-31-S1.ZIP › BPP-card13.JPG]

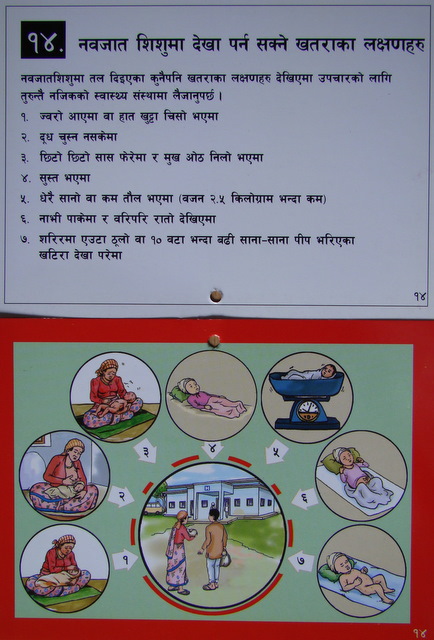

Supplement: Additional file 1 — Booklet cards. Reproductions of all 18 booklet cards are included in a zip file. [file 1471-2393-10-31-S1.ZIP › BPP-card14.JPG]

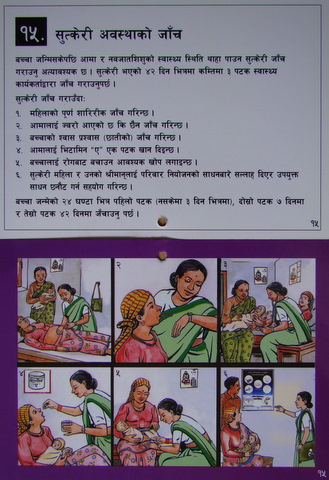

Supplement: Additional file 1 — Booklet cards. Reproductions of all 18 booklet cards are included in a zip file. [file 1471-2393-10-31-S1.ZIP › BPP-card15.JPG]

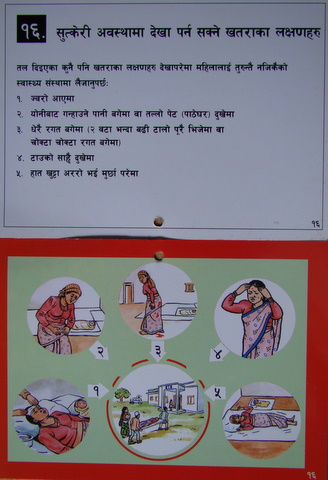

Supplement: Additional file 1 — Booklet cards. Reproductions of all 18 booklet cards are included in a zip file. [file 1471-2393-10-31-S1.ZIP › BPP-card16.JPG]

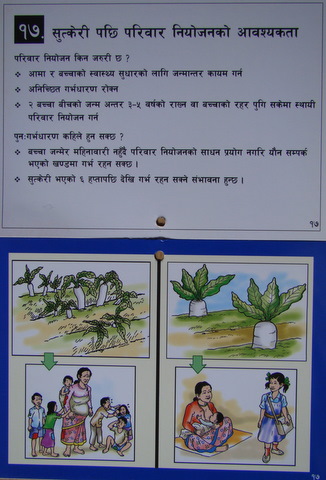

Supplement: Additional file 1 — Booklet cards. Reproductions of all 18 booklet cards are included in a zip file. [file 1471-2393-10-31-S1.ZIP › BPP-card17.JPG]
